# Systematic review of combined functional near-infrared spectroscopy and transcranial direct-current stimulation studies

**DOI:** 10.1117/1.NPh.7.2.020901

**Published:** 2020-06-25

**Authors:** Ronak Patel, Aleksander Dawidziuk, Ara Darzi, Harsimrat Singh, Daniel Richard Leff

**Affiliations:** St. Mary’s Hospital Campus, Imperial College London, Department of Surgery and Cancer, London, United Kingdom

**Keywords:** functional near-infrared spectroscopy, transcranial direct-current stimulation, systematic review

## Abstract

**Significance:** Combining transcranial direct-current stimulation (tDCS) with functional near-infrared spectroscopy (fNIRS) is a recent approach to exploring brain activation evoked by neurostimulation.

**Aim:** To critically evaluate studies combining tDCS and fNIRS and provide a consolidated overview of cortical hemodynamic responses to neurostimulation.

**Approach:** Key terms were searched in three databases (MEDLINE, EMBASE, and PsycINFO) with cross-referencing and works from Google Scholar also evaluated. All studies reporting on fNIRS-derived hemoglobin changes evoked by tDCS were included.

**Results:** Literature searches revealed 474 articles, of which 28 were included for final review (22 in healthy individuals: 9 involving rest and 13 with tasks; 6 in the clinical setting). At rest, an overall increase in cortical activation was observed in fNIRS responses at the site of stimulation, with evidence suggesting nonstimulated brain regions are also similarly affected. Conversely, during tasks, reduced cortical activation was observed during online stimulation. Offline and poststimulation effects were less consistent, as is the impact on clinical populations and their symptom correlation.

**Conclusion:** This review explores the methodological frameworks for fNIRS-tDCS evaluations and summarizes hemodynamic responses associated with tDCS in all populations. Our findings provide further evidence of the impact of tDCS on neuronal activation within functionally connected networks.

## 1 Introduction

Transcranial direct-current stimulation (tDCS) is a noninvasive neurostimulation method thought to modulate cortical activation that has recently gained a rapid rise within neuroscience research.^1^ Application of tDCS has revealed beneficial effects in patients with chronic pain syndromes^2^^–^^4^ and neuropsychiatric conditions,^5^^–^^10^ whereas for healthy individuals, tDCS has demonstrated performance gains in various cognitive^11^^–^^14^ and motor domains.^15^^–^^18^ However, results from published studies are far from conclusive, with some studies failing to corroborate otherwise observed effects.^19^^–^^21^ An increasingly accepted view within the tDCS research community is that interindividual variability has a significant influence on research findings with contributing factors including electrical field distribution,^22^ stimulation intensity,^23^ type of stimulation,^24^ and participant factors, such as age, anatomy, and presence of brain injury.^25^ These aspects are adding to the growing understanding of underlying neural mechanisms underpinning tDCS-led improvements.

Current knowledge of tDCS-induced neural changes stems from animal studies in which surface-positive current was observed to enhance neuronal firing and the size of evoked potentials.^26^ In humans, transcranial magnetic stimulation (TMS) has allowed for quantification of motor-cortical neuronal responses with the size of motor-evoked potentials (MEPs) corresponding to the excitability of the primary motor cortex (M1). Of note, tDCS has produced an increase in the size of MEPs^27^^,^^28^ during stimulation while additional studies have demonstrated the role of GABAergic and glutamatergic synaptic modulation in the poststimulation period.^29^^–^^31^ However, these studies largely focus on motor cortex changes as cortical excitability outside of this region cannot be easily measured. Hence, tDCS-induced neural changes in other brain regions are less well known, which has further prompted the need for investigation of concomitant stimulation and functional neuroimaging.

Studies have previously combined stimulation with neuroimaging methods, such as functional magnetic resonance imaging (fMRI),^32^^–^^35^ positron emission tomography (PET),^36^^,^^37^ and electroencephalography (EEG).^38^^,^^39^ However, fMRI may be susceptible to artifacts due to variable magnetic fields created with concurrent tDCS.^40^ Furthermore, it is expensive, precludes sufferers of claustrophobia, and has clear limitations in mobility for real-world tasks. Along with these factors, PET has the additional concern of radiotracer administration and radiation exposure. Functional near-infrared spectroscopy (fNIRS) is an indirect neuroimaging technique that is intrinsically independent of electrical stimulation by quantifying concentration changes in oxygenated (${\mathrm{HbO}}_{2}$), deoxygenated (HHb), and total (HbT) hemoglobin in real time. As well as being cost-effective, the technique has greater spatial resolution compared to EEG and heightened temporal resolution compared to fMRI.^41^^,^^42^ Of importance, fNIRS is relatively resistant to movement artifacts, and recent technological developments have introduced portable systems,^43^ creating the opportunity to implement the technology in real world scenarios.

**Fig. 1:**
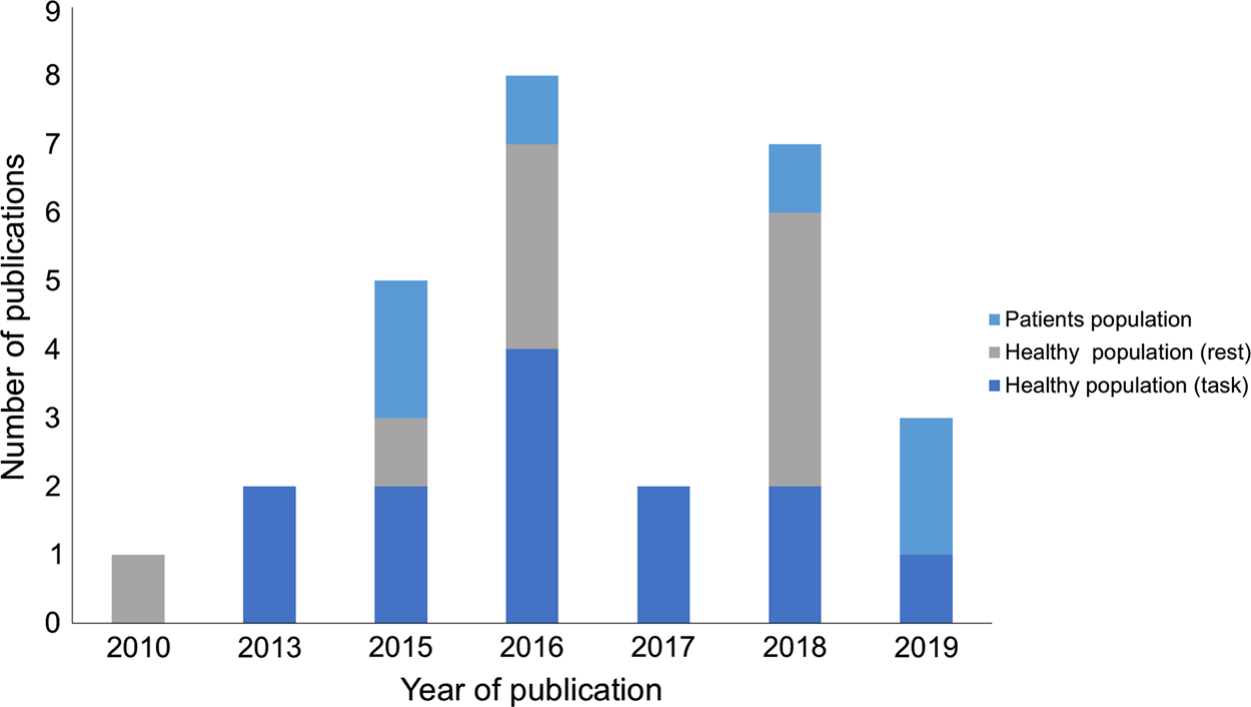
Number of publications utilizing a combined tDCS and fNIRS montage by year.

The advantage of combining tDCS with fNIRS is evidenced by a recent surge in publications employing a combined stimulation-neuroimaging experimental framework (Fig. 1), but despite the growing interest, there has been no systematic review of these studies to critically evaluate the impact of tDCS on fNIRS responses. Therefore, this article aims to explore the technical frameworks used in tDCS-fNIRS integration and provide a comprehensive summary of the impact of tDCS on changes to hemoglobin species and its implications for the underlying mechanistic effects of stimulation.

## 2 Methods

### 2.1 Search Strategy

An electronic search of EMBASE (1947 to July 2019), MEDLINE (1946 to July 2019), and PsycINFO (1806 to July 2019) was conducted with the following combinations of terms: (“transcranial direct current stimulation” OR “transcranial electric stimulation” OR “transcranial DC stimulation” OR “tDCS”) AND (“near-infrared spectroscopy” OR “near-infrared spectroscopy” OR “infrared spectroscopy” OR “functional near-infrared” OR “near infrared” OR “fNIRS” OR “NIRS” OR “diffuse optical imaging” OR “optical imaging” OR “optical topography” OR “cerebral oximetry”). Results were limited to studies involving human subjects and reported in English language. Additional records were identified through Google Scholar search and cross-referencing bibliographies of included studies. The last date for this literature search was July 12, 2019.

### 2.2 Eligibility Criteria

#### 2.2.1 Inclusion criteria

The publications were included in the review only if they met all of the following criteria:

1. Original experimental studies collecting data on human subjects.
2. Studies utilizing fNIRS and tDCS within the same experimental protocol.
3. Studies reporting the change in the concentration of hemoglobin species with tDCS

#### 2.2.2 Exclusion criteria

Works of nonexperimental nature (reviews, editorials, letters, and short surveys), dissertations, conference abstracts, and methodological papers not involving any human subjects were excluded. In addition, studies employing imaging other than fNIRS or stimulation techniques other than tDCS were not included.

### 2.3 Data Extraction

Potentially relevant studies were screened on the basis of their titles and abstracts by two authors (AD, RP). Full texts of the publications meeting the inclusion criteria were obtained and analyzed for eligibility. A summary of the articles included in the final review is detailed in Table 1. Data extracted from the included studies were recorded using Microsoft Excel for Mac Version 16.28 (Microsoft Corporation, Redmond, Washington). The following information was recorded: population characteristics, number of participants, protocol used, task employed, type of sham, tDCS and fNIRS setup, stimulation and imaging parameters and locations, and primary findings. Studies were analyzed for qualitative and quantitative changes in fNIRS-measured Hb species including ${\mathrm{HbO}}_{2}$, HHb, HbT, and ${\mathrm{Hb}}_{\mathrm{diff}}$ (${\mathrm{HbO}}_{2}-\mathrm{HHb}$). Reporting of raw values or summary statistics for Hb species changes was noted to be limited across many studies but is included where possible. Moreover, to provide a comprehensive overview of fNIRS responses, all authors were contacted to request original data for each study to facilitate a quantitative assessment. Based on heterogeneity of included studies, pooled statistical analysis of quantitative results was not possible.

### 2.4 Quality Assessment

To ensure thorough assessment of the selected articles, quality was independently assessed by two authors (RP and AD). The “Jadad Score”^44^ was applied to all sham-controlled studies. In the three studies that used more than one intervention arm (but not including sham), blinding was removed from the scoring system, giving a total possible score of three. It was not appropriate to apply this quality scoring method to the nine studies in which only one intervention was studied as there was no scope for randomization or blinding in these studies. Any disagreement regarding quality assessment was resolved through discussion with a senior author (DRL).

## 3 Results

### 3.1 Study Selection

**Fig. 2:**
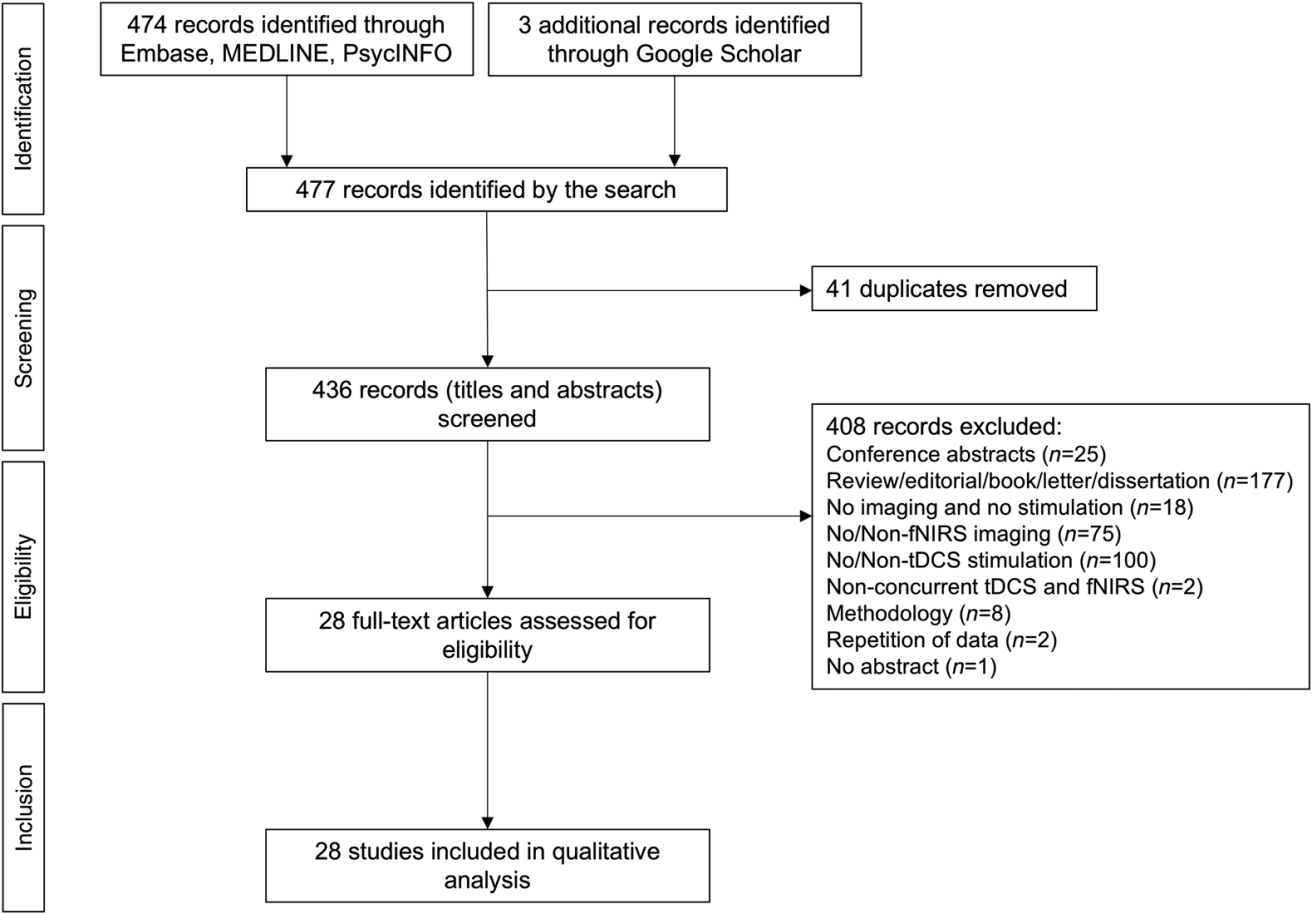
PRISMA flow diagram presenting the process of study selection.

Figure 2 shows the study selection process. After deduplication, 433 articles were identified from the initial search with three additional studies from Google Scholar and cross-referencing. Following screening and analysis against inclusion and exclusion criteria, a total of 408 publications were excluded leaving a total of 28 articles for final review.

### 3.2 Review Organization

Table 1 provides a summary of all 28 studies presented in this review. Selected works were assigned into three subcategories, as follows: healthy subjects at rest (n=9), healthy subjects performing tasks (n=13), and subjects with medical conditions (n=6). This review will first focus on evaluating protocols and technical aspects of combining tDCS with fNIRS in all of the selected studies. It will be followed by a subsequent analysis of methods and findings presented by publications according to above-mentioned assortment.

**Table 1:** Studies investigating the effects of tDCS on fNIRS-measured response. L, left; R, right; PFC, prefrontal cortex; M1, primary motor cortex.

Reference	Population	N	Task	Concurrent tDCS/fNIRS	Stimulation		fNIRS measurement	
					Location	Parameters	Location	Parameters
Merzagora et al., 2010^56^	Healthy	12	—	—	Bilateral PFC	1 mA, $0.029\;\mathrm{mA}/{\mathrm{cm}}^{2}$	Bilateral PFC	16 channels
								4 emitters, 10 detectors
						10 or 15 min		
Bhutta et al., 2016^57^	Healthy	3	—	—	Bilateral PFC	1 mA, $0.029\;\mathrm{mA}/{\mathrm{cm}}^{2}$	Bilateral PFC	16 channels
						10 min		4 emitters, 10 detectors
Yaqub et al., 2018^50^	Healthy	15	—	✓	R PFC	1 mA, $1.275\;\mathrm{mA}/{\mathrm{cm}}^{2}$	Bilateral PFC	32 channels
								14 optodes
						10 min		
Yan et al., 2015^64^	Healthy	5	—	✓	L M1	1.5 mA, $0.043\;\mathrm{mA}/{\mathrm{cm}}^{2}$	R M1	7 channels
								3 emitters, 3 detectors
						5 min		
Sood et al., 2016^49^	Healthy	5	—	✓	L M1	2 mA, $0.637\;\mathrm{mA}/{\mathrm{cm}}^{2}$	Bilateral M1	16 channels
								12 emitters, 4 detectors
						10 min		
Takai et al., 2016^65^	Healthy	7	—	✓	Bilateral M1	1 mA, $0.026\;\mathrm{mA}/{\mathrm{cm}}^{2}$	Bilateral M1	34 channels
								12 emitters, 12 detectors
						20 min		
Muthalib et al., 2018^51^	Healthy	13	—	✓	L M1	2 mA, $0.037\;\mathrm{mA}/{\mathrm{cm}}^{2}$	Bilateral M1	16 channels
						10 min		12 emitters, 4 detectors
Cao et al., 2018^a^^70^	Healthy	13	—	✓	Bilateral frontal	1: 0.5 mA, $0.02\;\mathrm{mA}/{\mathrm{cm}}^{2}$	PFC + frontal	83 channels
						2.6 min		
								26 emitters, 28 detectors
						2: 1 mA, $0.04\;\mathrm{mA}/{\mathrm{cm}}^{2}$		
						8 min		
Cao and Liu, 2018^b^^69^	Healthy	13	—	✓	Bilateral frontal	1: 0.5 mA, $0.02\;\mathrm{mA}/{\mathrm{cm}}^{2}$	PFC + frontal	83 channels
								26 emitters, 28 detectors
						2.6 min		
						2: 1 mA, $0.04\;\mathrm{mA}/{\mathrm{cm}}^{2}$		
						8 min		
Khan, 2013^71^	Healthy	8	✓	✓	Bilateral M1	2 mA, $0.08\;\mathrm{mA}/{\mathrm{cm}}^{2}$	Bilateral M1	84 channels
								32 emitters, 16 detectors
						15 min		
Muthalib et al., 2013^66^	Healthy	15	✓	✓	R M1	2 mA, $0.083\;\mathrm{mA}/{\mathrm{cm}}^{2}$	R PFC	3 emitters, 2 detectors
						2×10 min		
Muthalib et al., 2016^45^	Healthy	8	✓	✓	L M1	2 mA, $0.637\;\mathrm{mA}/{\mathrm{cm}}^{2}$	Bilateral M1	16 channels
						20 min		12 emitters, 4 detectors
Radel et al., 2017^46^	Healthy	22	✓	✓	R PFC + M1	2 mA, $4\;\mathrm{mA}/{\mathrm{cm}}^{2}$	R PFC + M1	2 channels
						10 min		2 emitters, 1 detector
Besson et al., 2019^47^	Healthy	15	✓	✓	L M1	2 mA, $0.637\;\mathrm{mA}/{\mathrm{cm}}^{2}$	L M1	4 channels
						20 min		2 emitters, 2 detectors
Jones et al., 2015^58^	Healthy	Exp1 24 Exp2 20	✓	—	L PFC	1.5 mA, $0.042\;\mathrm{mA}/{\mathrm{cm}}^{2}$	L PFC	3 channels
								1 emitter, 3 detectors
						10 min		
McKendrick et al., 2015^48^	Healthy	unknown	✓	✓	R PFC	1 mA	Bilateral PFC	16 channels
								4 emitters, 10 detectors
Stephens and Berryhill, 2016^59^	Healthy	90	✓	—	R PFC	1: 1 mA, $0.029\;\mathrm{mA}/{\mathrm{cm}}^{2}$	Bilateral PFC	14 channels
						2: 2 mA, $0.057\;\mathrm{mA}/{\mathrm{cm}}^{2}$		
						15 min		
Ehlis et al., 2016^60^	Healthy	46	✓	—	Bilateral frontal	1 mA, $0.029\;\mathrm{mA}/{\mathrm{cm}}^{2}$	Bilateral frontal	44 channels
								16 emitters, 14 detectors
						20 min		
Herrmann et al., 2017^72^	Healthy	61	✓	✓	Bilateral PFC	1 mA, $0.029\;\mathrm{mA}/{\mathrm{cm}}^{2}$	Bilateral PFC + frontal	52 channels
						26 min		15 emitters, 16 detectors
Borragan et al., 2018^67^	Healthy	22	✓	✓	Bilateral PFC	1.5 mA, $0.075\;\mathrm{mA}/{\mathrm{cm}}^{2}$	Bilateral frontal	6 channels
								2 emitters, 6 detectors
						25 min		
Giovannella et al., 2018^54^	Healthy	20	✓	✓	Bilateral frontal	1 mA, $0.884\;\mathrm{mA}/{\mathrm{cm}}^{2}$	Bilateral frontal	2 channels
								2 emitters, 2 detectors
						10 min		
Choe et al., 2016^52^	Healthy	32	✓	✓	Bilateral PFC + M1	2 mA, $0.04\;\mathrm{mA}/{\mathrm{cm}}^{2}$	Bilateral PFC + M1	20 channels
								8 emitters, 8 detectors
						60 min		
Dutta et al., 2015^53^	Disease	4	—	✓	Cz	0.5 mA, $0.053\;\mathrm{mA}/{\mathrm{cm}}^{2}$	Cz	4 channels
								1 emitter, 4 detectors
						15×30 s		
Jindal et al., 2015^55^	Disease	5	—	✓	Bilateral PFC	$0.053\;\mathrm{mA}/{\mathrm{cm}}^{2}$	Bilateral PFC	2 emitters, 2 detectors
						15×30 s		
Kroczek et al., 2016^68^	Disease	25	✓	✓	Bilateral PFC	2 mA, $0.057\;\mathrm{mA}/{\mathrm{cm}}^{2}$	Bilateral PFC	13 emitters, 12 detectors
						15 min		
Narita et al., 2018^61^	Disease	28	—	—	Bilateral PFC	2 mA, $0.057\;\mathrm{mA}/{\mathrm{cm}}^{2}$	Bilateral PFC	52 channels
						20 min		
Li et al., 2019^62^	Disease	22	✓	—	Bilateral PFC	2 mA	Bilateral PFC	20 channels
								8 emitters, 7 detectors
						20 min		
Verma et al., 2019^63^	Disease	1	✓	—	R PFC	2 mA, $0.044\;\mathrm{mA}/{\mathrm{cm}}^{2}$	Bilateral PFC	20 channels
								8 emitters, 8 detectors
						20 min		

a Biomed. Opt. Express.

b Neurophotonics.

### 3.3 Technical Considerations of Combined fNIRS and tDCS

**Fig. 3:**
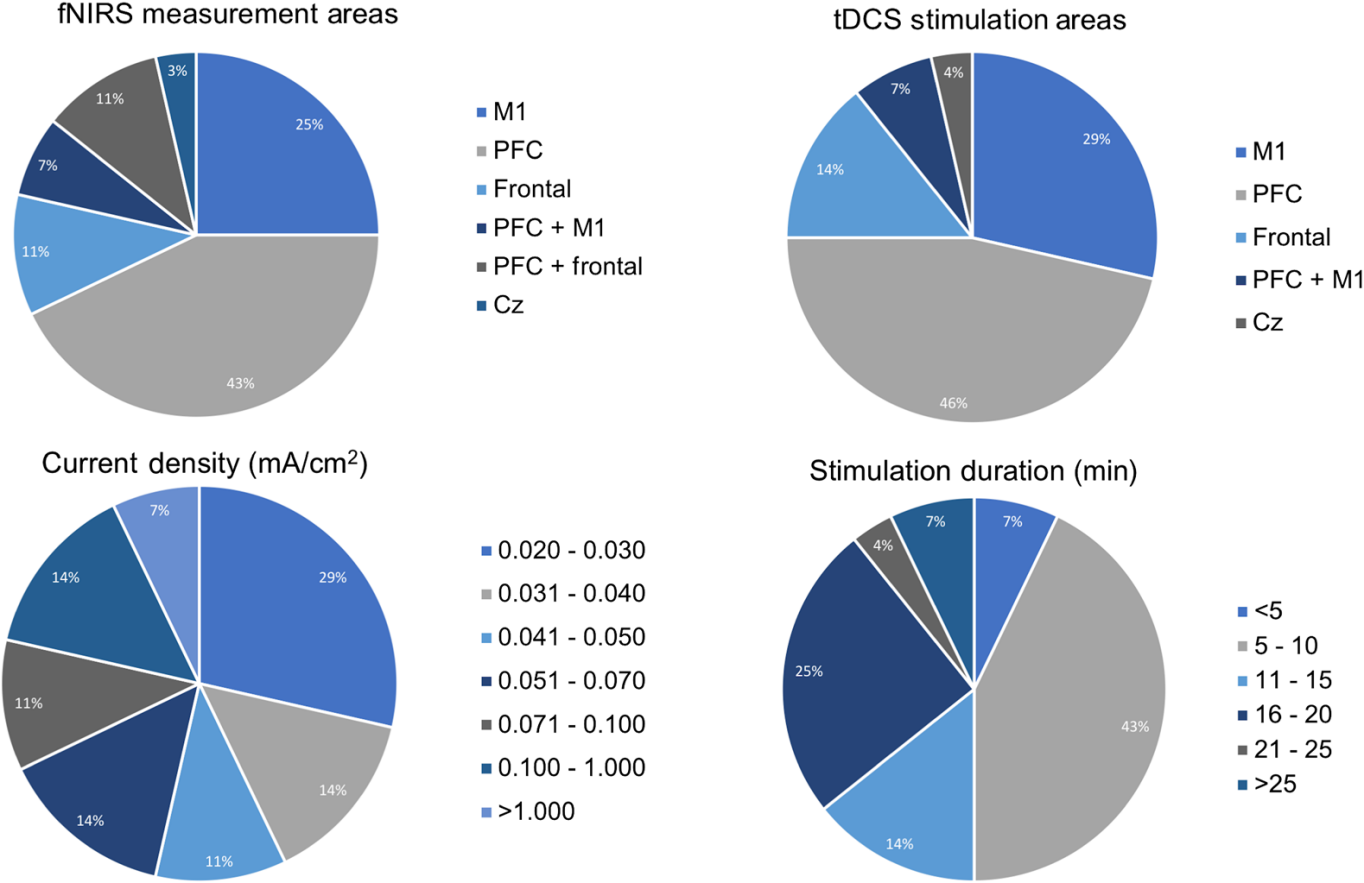
Location of fNIRS monitoring, location of stimulation, current density, and stimulation duration utilized in the studies (n=28). M1, primary motor cortex; PFC, prefrontal cortex

Since combining tDCS with fNIRS to monitor changes in brain activation is novel, the various methodological strategies for data acquisition are highly informative. Stimulation and fNIRS parameters revealed considerable heterogeneity among the studies with Fig. 3 illustrating the various locations, stimulation intensities, and durations of stimulation used. A high-definition tDCS (HD-tDCS) montage was utilized in eight studies.^45^^–^^52^ fNIRS montages ranged from 1- to 84-channel systems with five investigations additionally incorporating EEG into their fNIRS/tDCS montage setup.^49^^,^^52^^–^^55^ These variations in methodology are unsurprising given that it is appropriate to vary stimulation-hemodynamic acquisition protocol according to the proposed scientific question under study. Of course, it is absolutely appropriate to localize stimulation and fNIRS measurement to the motor cortex or the prefrontal cortex if investigating the impact in stroke survivors or depression, respectively. However, other variations in setup (including current density, duration of stimulation, repeated sessions, and variation in optode configuration) contribute further methodological heterogeneity, which can make it challenging to derive consistent conclusions.

**Fig. 4:**
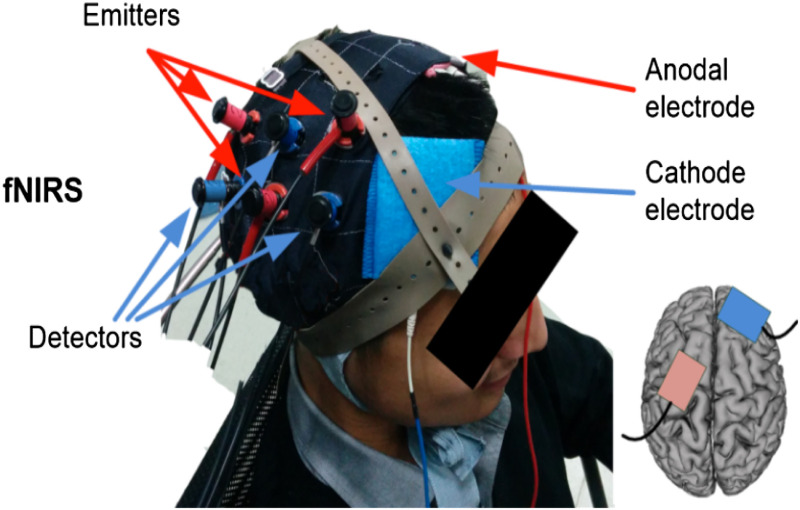
Example of concurrent fNIRS-tDCS setup using distant locations for tDCS and fNIRS to allow simultaneous use of both. Adapted with permission from Ref. 64.

One of the main challenges with tDCS-fNIRS integration lies within the technical framework for equipment setup. In the majority of studies, 22 out of 28, hemodynamic changes were recorded from the exact same location as stimulation was conducted, and concurrent stimulation and fNIRS measurement were performed in 20 of 28 studies (Table 1). Combining tDCS electrodes with fNIRS optodes over the same scalp location presents researchers with a practical challenge of costimulation with hemodynamic data acquisition. Some studies avoid this difficulty altogether by avoiding concurrent stimulation and fNIRS monitoring,^56^^–^^63^ as shown in Fig. 4. However, it is of considerable interest to study cortical changes during the stimulation process to gain further insight into changes in cortical hemodynamics during tDCS. Instead, certain studies describe measuring fNIRS responses in the hemisphere contralateral to stimulation,^64^^,^^65^ or in a different region within the same hemisphere.^66^ Another approach was to measure responses in the same general brain region, but not in the exact same surface location.^55^^,^^67^^,^^68^ The remaining studies used a variety of methods to integrate tDCS electrodes and fNIRS optodes over an identical surface location simultaneously. The majority utilized commercial devices that combine tDCS with fNIRS within a premade headcap and precludes any further technical equipment alterations by the investigator.^45^^–^^47^^,^^49^^,^^51^^,^^52^ However, certain investigators created custom-built assimilation by placing fNIRS optodes through the rubber tDCS electrode pads using either a hole-punching,^69^^–^^71^ drilling,^72^ or unspecified^53^ method. Others have elected to simultaneously hold fNIRS optodes and tDCS electrodes in place using a specially designed headset apparatus.^50^^,^^54^

### 3.4 fNIRS Responses in the Healthy Population at Rest

A total of nine studies investigated changes in cortical hemodynamics following tDCS to the cerebral cortex of healthy individuals at rest using fNIRS (Table 2). The rest period was reasonably standardized across six studies^49^^,^^50^^,^^56^^,^^57^^,^^64^^,^^65^ placing the subject in a seated position. Two studies asked subjects to keep their eyes closed^69^^,^^70^ and two studies to keep eyes open.^49^^,^^50^ One study instructed subjects to keep a fixed gaze on a screen^64^ and the remaining studies did not specify eye commands.

**Table 2:** Studies investigating the effects of tDCS on fNIRS-measured response in healthy population at rest. HD, high definition; PFC, prefrontal cortex; M1, primary motor cortex; SOR, supraorbital region; SMA, supplementary motor area; SMC, sensorimotor cortex; S1, primary sensory cortex; L, left; R, right.

Reference	N	Concurrent tDCS/fNIRS	Stimulation protocol	tDCS parameters	Stimulation area	fNIRS parameters	Measurement area	Overall fNIRS response
Merzagora et al., 2010^56^	12	—	Active/sham (two session crossover)	1 mA, $0.029\;\mathrm{mA}/{\mathrm{cm}}^{2}$, 10 or 15 min	Bilateral PFC	16 channels, 4 emitters, 10 detectors	Bilateral PFC	↑ ${\mathrm{HbO}}_{2}$ post tDCS versus sham
								Greater increase under anode
								Longer-lasting effect with 15 min stimulation versus 10 min
Bhutta et al., 2016^57^	3	—	Active	1 mA, $0.029\;\mathrm{mA}/{\mathrm{cm}}^{2}$, 10 min	Bilateral PFC	16 channels, 4 emitters, 10 detectors	Bilateral PFC	↑ ${\mathrm{HbO}}_{2}$ and ↓ HHb post tDCS versus Baseline
Yaqub et al., 2018^50^	15	✓	Active	1 mA, $1.275\;\mathrm{mA}/{\mathrm{cm}}^{2}$, 10 min	HD to R-PFC	32 channels, 14 optodes	Bilateral PFC	↑ ${\mathrm{HbO}}_{2}$ during and post tDCS
								↑ connectivity during and post tDCS, ↑ connectivity in R PFC versus L PFC
								↑ interhemispheric connectivity
Yan et al., 2015^64^	5	✓	Active	1.5 mA, $0.043\;\mathrm{mA}/{\mathrm{cm}}^{2}$, 5 min	L-M1 + R SOR	7 channels, 3 emitters, 3 detectors	R M1	↔ ${\mathrm{HbO}}_{2}$ during or post tDCS
								↓ connectivity in contralateral cortex
Sood et al., 2016^49^	5	✓	Active	2 mA, $0.637\;\mathrm{mA}/{\mathrm{cm}}^{2}$, 10 min	HD to L-SMC	16 channels, 12 emitters, 4 detectors	Bilateral SMC	↑ ${\mathrm{HbO}}_{2}$ and ↓ HHb during tDCS
Takai et al., 2016^65^	7	✓	Anodal/cathodal/sham (3 session crossover)	1 mA, $0.026\;\mathrm{mA}/{\mathrm{cm}}^{2}$, 20 min	R-M1 + L SOR	34 channels, 12 emitters, 12 detectors	L-PMC, SMA, L-SMC	↓ ${\mathrm{HbO}}_{2}$ with anodal and cathodal versus sham in PMC, SMA, M1.
								↑ ${\mathrm{HbO}}_{2}$ in S1 in anodal versus cathodal/sham
Muthalib et al. 2018^51^	13	✓	2 × identical active/sham (three session crossover)	2 mA, $0.037\;\mathrm{mA}/{\mathrm{cm}}^{2}$, 10 min	HD to L-M1	16 channels, 12 emitters, 4 detectors	L + R hemispheres	↑ ${\mathrm{HbO}}_{2}$ in L-hemisphere, greater within region of stimulation.
								↑ ${\mathrm{HbO}}_{2}$ with sham but of lower amplitude
Cao et al., 2018^a^^70^	13	✓	2 × active stimulation periods at different current intensity	1: 0.5 mA, $0.02\;\mathrm{mA}/{\mathrm{cm}}^{2}$, 2.6 min	L-Broca’s area + R SOR	83 channels, 26 emitters, 28 detectors	Bilateral Broca’s, Wernicke’s areas, PFC	↑ in L and R Broca’s area information outflow during and post both stimulation intensities
				2: 1 mA, $0.04\;\mathrm{mA}/{\mathrm{cm}}^{2}$, 8 min				
Cao and Liu, 2018^b^^69^	13	✓	2 × active stimulation periods at different current intensity	1: 0.5 mA, $0.02\;\mathrm{mA}/{\mathrm{cm}}^{2}$, 2.6 min	L-Broca’s area + R SOR	83 channels, 26 emitters, 28 detectors	Bilateral Broca’s, Wernicke’s areas, PFC	↑ local and ↓ remote functional connectivity induced by tDCS
				2: 1 mA, $0.04\;\mathrm{mA}/{\mathrm{cm}}^{2}$, 8 min				

a *Biomed. Opt. Express*.

b *Neurophotonics*.

#### 3.4.1 Prefrontal stimulation

**Fig. 5:**
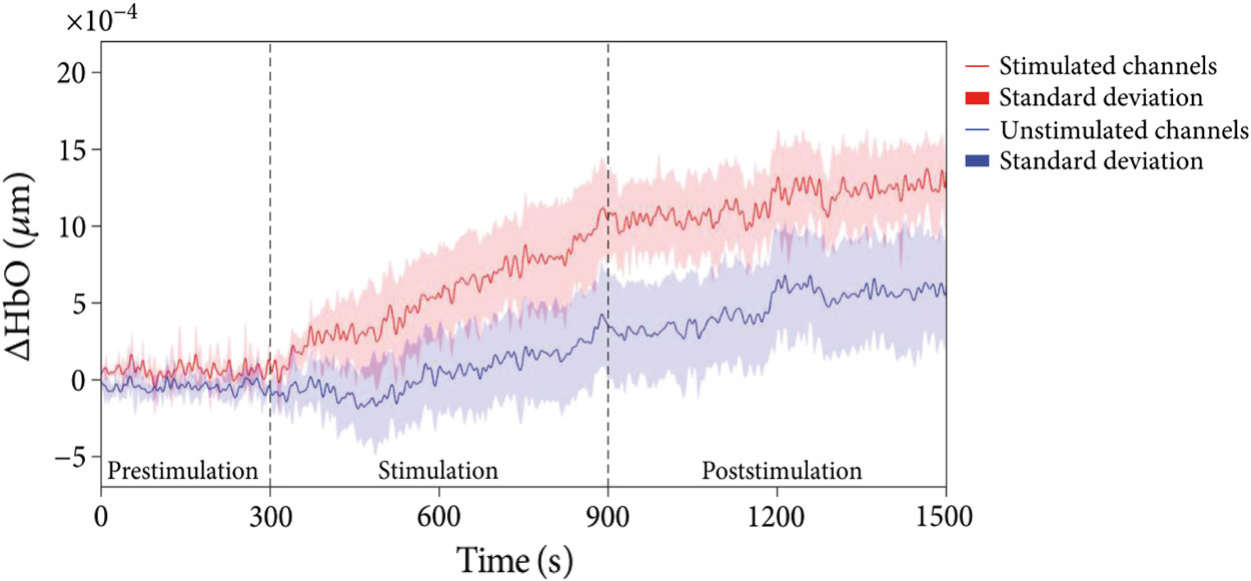
Representative example of fNIRS Hb time series analysis during rest. Following stimulation there is an immediate significant increase in ${\mathrm{HbO}}_{2}$ compared to unstimulated regions. Subsequently, the increased ${\mathrm{HbO}}_{2}$ trace is maintained poststimulation. Adapted with permission from Ref. 50.

Among all studies, a general tendency for tDCS to increase ${\mathrm{HbO}}_{2}$ was observed. This was consistent across all three studies applying PFC stimulation,^50^^,^^56^^,^^57^ all of which recorded fNIRS activation within the same region as stimulation. Two of these^56^^,^^57^ applied 1-mA bilateral PFC stimulation (anode left PFC, Fp1 and cathode right PFC, Fp2) and demonstrated a peak in ${\mathrm{HbO}}_{2}$ in the bilateral PFC region ∼4  min after the end of stimulation before returning to baseline levels. This was more pronounced under the left anode and with 15 min of stimulation compared to 10 min.^56^ An increase in ${\mathrm{HbO}}_{2}$ in the bilateral PFC region was also observed during 1-mA HD stimulation to the right PFC, which was maintained poststimulation as shown in Fig. 5 (mean ${\mathrm{HbO}}_{2}$ in right PFC stimulated channels: $6.90647\times 10^{-4}$ versus mean ${\mathrm{HbO}}_{2}$ in all other unstimulated channels across the bilateral PFC: $1.96703\times 10^{-4}$) along with increased intra- and interhemispheric connectivity.^50^ A placebo group was included in only one PFC stimulation study^56^ in which no such ${\mathrm{HbO}}_{2}$ changes were observed across the bilateral PFC region with sham bilateral PFC stimulation. HHb was only analyzed in one study demonstrating a decrease in HHb alongside the increase in ${\mathrm{HbO}}_{2}$ in the bilateral PFC region.^57^ Notably, HHb was not analyzed in the remaining two studies^50^^,^^56^ due to a “lack of effect,” presumably as no significant changes in HHb were observed.

#### 3.4.2 Motor cortex stimulation

Similar findings were observed with motor cortex stimulation. Sood et al.^49^ applied 2-mA HD stimulation to the left motor cortex and, after an initial drop, observed an overall increase in ${\mathrm{HbO}}_{2}$ coupled with a decrease in HHb in the sensorimotor cortex bilaterally. The study does not clearly differentiate between the laterality of these responses, and it is possible that this change may be referring to the left cortex, ipsilateral to stimulation, and thus is in keeping with the findings of PFC stimulation. Two additional task-based studies^46^^,^^71^ recorded fNIRS responses at rest (prior to any task) with motor cortex stimulation. Following 2-mA stimulation to the bilateral motor cortices, regardless of anodal/cathodal polarity, there was an increase in ${\mathrm{HbO}}_{2}$ across the bilateral sensorimotor cortex.^71^ A similar observation was demonstrated compared to baseline following 2-mA anodal HD stimulation to the right M1.^46^ These findings are supported by a well-designed study^51^ that repeated (for reproducibility of results) two identical 2-mA anodal HD stimulation sessions to the left M1, alongside a sham stimulation session. fNIRS responses were measured across the scalp in both hemispheres and were similar for the ipsilateral (left) cortex (initial slight decrease in ${\mathrm{HbO}}_{2}$, followed by increase), and greatest within the region of left HD M1 stimulation. A similar response was observed in the sham group but of far lower amplitude and with more rapid return to baseline. In this same study,^51^ no significant changes were observed in ${\mathrm{HbO}}_{2}$ levels from baseline in the contralateral (right) cortical hemisphere and no differences in ${\mathrm{HbO}}_{2}$ identified between active and sham stimulation. Similarly, Yan et al.^64^ administered 1.5-mA left anodal M1 stimulation, although for only 5 min and observed no overall change in ${\mathrm{HbO}}_{2}$ in the right parietal cortex. However, reduced contralateral (right) connectivity during left anodal M1 tDCS was demonstrable, suggesting that stimulation could affect the contralateral brain region.^64^ This contralateral lateralized effect was confirmed in another study^65^ in which 1-mA right anodal and cathodal M1 stimulation resulted in a decrease in ${\mathrm{HbO}}_{2}$ in the left PMC, SMA, and M1 compared to sham.

In a series of studies, Cao et al.^69^^,^^70^ did not report on the changes in Hb subspecies, but rather focused on neural connectivity, with Broca’s area becoming an outflow information “hotspot” during and after active 0.5 and 1.0 mA anodal tDCS to left Broca’s area, as well as increased connectivity between left Broca’s area and the regions immediately surrounding it.

### 3.5 Task-Evoked fNIRS Responses in the Healthy Population

The effects of tDCS on cognitive and motor task-evoked fNIRS responses in the healthy population were explored in 13 studies, as summarized in Table 3.

**Table 3:** Studies investigating the effects of tDCS on fNIRS-measured response in healthy population during task performance. MIVC, maximal isometric volumetric contraction; WM, working memory; HD, high definition; PFC, prefrontal cortex; dlPFC, dorsolateral prefrontal cortex; M1, primary motor cortex; SOR, supraorbital region; SMC, sensorimotor cortex; L, left; R, right.

Reference	N	Task	Concurrent tDCS/fNIRS	Stimulation protocol	tDCS parameters	Stimulation area	fNIRS parameters	Measurement area	Overall fNIRS response	
Motor										
Khan 2013^71^	8	Wrist flexion—50% of MIVC	✓	Active/reverse polarity (two session crossover)	2 mA, $0.08\;\mathrm{mA}/{\mathrm{cm}}^{2}$, 15 min	Bilateral M1	84 channels, 32 emitters, 16 detectors	Bilateral SMC	↑ interhemispheric connectivity and ↑ activation under anodal electrode during and post-tDCS	
Muthalib et al., 2013^66^	15	Elbow flexion—30% of MIVC	—	Active/sham(two session crossover)	2 mA, $0.083\;\mathrm{mA}/{\mathrm{cm}}^{2}$, 2×10 min	R-M1+ R shoulder	3 emitters, 2 detectors	R PFC	↑ ${\mathrm{HbO}}_{2}$ and ↓ HHb postactive and sham tDCS—no difference between the two	
Muthalib et al., 2016^45^	8	Finger sequence task	✓	Active	2 mA, $0.637\;\mathrm{mA}/{\mathrm{cm}}^{2}$, 20 min	HD to the L-SMC	16 channels, 12 emitters, 4 detectors	Bilateral SMC	↑ ${\mathrm{HbO}}_{2}$ increase in L-SMC during tDCS versus baseline	
									Smaller magnitude ↓ HHb in L + R-SMC during and post tDCS versus baseline	
Radel et al., 2017^46^	22	Elbow flexion—35% of MIVC	✓	PFC/M1/sham (three session crossover)	2 mA, $4\;\mathrm{mA}/{\mathrm{cm}}^{2}$, 10 min	HD to R-PFC/R-M1	2 channels, 2 emitters, 1 detector	R-PFC, R-M1	↓ ${\mathrm{HbO}}_{2}$ in PFC stimulation versus sham and M1stimulation	
Besson et al., 2019^47^	15	Single finger opposition	✓	Online task/offline task/sham (three session crossover)	2 mA, $0.637\;\mathrm{mA}/{\mathrm{cm}}^{2}$, 20 min	HD to L-M1	4 channels, 2 emitters, 2 detectors	L-M1	Online: during task:	Offline: during task:
									↑ HHb versus Offline	↔ HHb/Hbdiff versus baseline/sham
									↓ Hbdiff versus baseline	
									–post task	–post task
									↓ HHb versus baseline/sham	↓ HHb versus baseline
									↑ Hbdiff versus baseline/sham	↔ Hbdiff versus baseline/sham
Cognitive										
McKendrick et al., 2015^48^	Unknown	Spatial WM task	✓	2×sham and 2×active (four stimulation periods in one session)	1 mA	HD to R-PFC	16 channels, 4 emitters, 10 detectors	Bilateral PFC	↓ activation with active tDCS versus sham.	
									Presumably through ↓ ${\mathrm{HbO}}_{2}$ and ↑ HHb	
Jones et al., 2015^58^	Exp1: 24	WM task with strategy instruction		Active/sham (two session crossover)	1.5 mA, $0.043\;\mathrm{mA}/{\mathrm{cm}}^{2}$, 10 min	L-PFC + R cheek	3 channels, 1 emitter, 3 detectors	L-PFC	Exp1: ${\mathrm{HbO}}_{2}$ ↑ with active tDCS, greatest during strategy use and in high WM group	
	Exp2: 20	WM task with differing financial incentive							Exp2: ${\mathrm{HbO}}_{2}$ ↑ in low WM versus high WM group with active tDCS. But no diff versus sham	
Stephens and Berryhill, 2016^59^	90 (elderly)	WM task		Active1/active2/ sham (parallel). Five consecutive sessions	1: 1 mA, $0.029\;\mathrm{mA}/{\mathrm{cm}}^{2}$ 2: 2 mA, $0.057\;\mathrm{mA}/{\mathrm{cm}}^{2}$ 15 min	R-PFC + L cheek	14 channels	Bilateral PFC	${\mathrm{HbO}}_{2}$ ↓ correlated with better performance in two-back task., regardless of tDCS condition	
Ehlis et al., 2016^60^	46	Verbal fluency task		Anodal or cathodal/sham (two session crossover)	1 mA, $0.029\;\mathrm{mA}/{\mathrm{cm}}^{2}$, 20 min	L-Broca’s area + R SOR	44 channels, 16 emitters, 14 detectors)	Bilateral frontotemporal area	${\mathrm{HbO}}_{2}$ ↑ with anodal tDCS versus sham	
									${\mathrm{HbO}}_{2}$ ↓ with cathodal tDCS versus sham (trend)	
Herrmann et al., 2017^72^	61	Verbal Fluency Task	✓	Active/reverse polarity/sham (parallel)	1 mA, $0.029\;\mathrm{mA}/{\mathrm{cm}}^{2}$, 26 min	Bilateral dlPFC	52 channels, 15 emitters, 16 detectors	Bilateral PFC	↑ ${\mathrm{HbO}}_{2}$ and ↓ HHb in FTC overall. No difference between groups during VFT	
Borragan et al., 2018^67^	22	WM task	✓	Active/sham (two session crossover)	1.5 mA, $0.075\;\mathrm{mA}/{\mathrm{cm}}^{2}$, 25 min	L-dlPFC + R forearm	6 channels, 2 emitters, 6 detectors)	Bilateral frontal	Immediately postfatiguing task:	
									–L hemi: ↑ ${\mathrm{HbO}}_{2}$ in L hemi versus baseline.	
									–R hemi: ↓ ${\mathrm{HbO}}_{2}$ versus sham	
									Postperiod:	
									L hemi: ↓ ${\mathrm{HbO}}_{2}$ versus baseline and sham	
Giovannella et al., 2018^54^	20	Visual task	✓	Anodal/cathodal/sham (three session crossover)	1 mA, $0.884\;\mathrm{mA}/{\mathrm{cm}}^{2}$, 10 min	L-frontal + R-parieto-occipital lobe	2 channels, 2 emitters, 2 detectors	Bilateral frontal cortex	L hemi only:	
									${\mathrm{HbO}}_{2}$ ↑ during and postanodal and cathodal versus baseline and sham.	
									HHb ↓ during and postanodal and cathodal versus baseline and cathodal versus sham	
Motor cognition										
Choe et al., 2016^52^	32	Cognitive and motor (WM tasks, flight stimulator)	✓	PFC/M1/sham (parallel).	2 mA, $0.04\;\mathrm{mA}/{\mathrm{cm}}^{2}$, 60 min	HD to L-M1 or R-dlPFC	20 channels, 8 emitters, 8 detectors	L-M1, R-dlPFC	Easy-landing task:	
				Four consecutive sessions						
									dlPFC stim: ↓ ${\mathrm{HbO}}_{2}$ in dlPFC and M1. Also observed in sham but with smaller magnitude	
									M1 stim: ↓ ${\mathrm{HbO}}_{2}$ and HHb versus ↑ ${\mathrm{HbO}}_{2}$ and HHb in sham	
									N-back task:	
									M1 stim: ↓ ${\mathrm{HbO}}_{2}$ in dlPFC, not observed in sham	

#### 3.5.1 Motor tasks

##### Online stimulation

The impact of tDCS on fNIRS responses during a motor task was described in five studies.^45^^–^^47^^,^^66^^,^^71^ tDCS was administered concurrently (online) with the task in four of these.^45^^–^^47^^,^^71^ These studies all identified reduced cortical activation, for example, Radel et al.^46^ observed an overall decrease in ${\mathrm{HbO}}_{2}$ with 2-mA anodal HD stimulation to the right M1. Furthermore, during 2-mA anodal HD stimulation to the left M1, either a smaller magnitude decrease in HHb in the bilateral SMC,^45^ or an overall decrease in ${\mathrm{Hb}}_{\mathrm{diff}}$ in the left M1 region was observed compared to baseline responses ($\mathrm{pre}=1.42259\times 10^{-5}\;\mu M$ versus $\text{during}=7.87907\times 10^{-6}\;\mu M$) (but not in sham stimulation).^47^ Conversely, one of these studies^45^ also demonstrates greater ${\mathrm{HbO}}_{2}$ in the stimulated left M1 suggestive of increased cortical activation. Although the authors argue that this was potentially due to increased skin blood flow, another online study^71^ detected increased activation but with short channel separation to filter out unrelated hemodynamic changes. Unfortunately, neither of these two studies^45^^,^^71^ included a sham group for comparison.

fNIRS responses in the postonline stimulation period were more varied. As per their findings during stimulation, Muthalib et al.^45^ observed a significantly smaller reduction in HHb (reduced activation) compared to baseline task-evoked responses in the bilateral sensorimotor cortex (left SMC: pre=−0.38Δ  μM versus post=−0.27Δ  μM; right SMC: pre=−0.34Δ  μM versus post=−0.28Δ  μM) following anodal HD left M1 stimulation. However, Khan^71^ demonstrated increased activation in the anodal region regardless of polarity in 2-mA dual motor stimulation compared to baseline task responses. This led to a persistence of interhemispheric connections with anode on ipsilateral side or a reduction in activation and intrahemispheric connectivity with the cathode placed ipsilaterally. This is supported by the only sham-controlled study,^47^ which examined left M1 fNIRS responses following 2-mA anodal HD stimulation to the left M1. The authors demonstrated increased activation indexed from a decrease in HHb compared to baseline ($\mathrm{pre}=-3.71899\times 10^{-6}\;\mu M$ versus $\text{post}=-5.64891\times 10^{-6}\;\mu M$) and compared to sham (active tDCS $\text{post}=-5.64891\times 10^{-6}\;\mu M$ versus sham $\text{post}=-3.64507\times 10^{-6}\;\mu M$) and also an increase in ${\mathrm{Hb}}_{\mathrm{diff}}$ compared to both baseline rest and sham stimulation.

##### Offline stimulation

Two studies^47^^,^^66^ examined an offline stimulation protocol with tDCS administered prior to a motor task stimulus. Both studies demonstrated an increase in activation (increase ${\mathrm{HbO}}_{2}$ and decrease HHb) poststimulation compared to baseline responses either in the same stimulation region (left M1)^47^ or in distant but ipsilateral regions (right PFC following anodal HD right M1 stimulation).^66^ However, these changes were not significantly different from sham stimulation groups.

#### 3.5.2 Cognitive tasks

##### Online stimulation

tDCS-modulated brain activation evoked by cognitive tasks was investigated in seven studies, all of which utilized sham stimulation protocols to assess effectiveness of tDCS. An online (task with stimulation) protocol was utilized in four studies^48^^,^^59^^,^^67^^,^^72^ with the general trend supporting a reduction in cortical activation. This was observed in the bilateral PFC during 1-mA anodal HD right PFC stimulation compared to sham stimulation during a spatial working memory (WM) task.^48^ Within this region, only a reduction in right dorsolateral and dorsomedial PFC activation specifically demonstrated a correlation to improved task performance. Immediately following online 1.5-mA anodal stimulation of the left PFC, an initial increase in ipsilateral frontal cortical oxygenation (cerebral oxygen exchange: $\mathrm{pre}=-3.17\times 10^{14}$ versus $\text{post}=-4\times 10^{14}$; no units) was followed by a decrease 20 min later ($\text{post}\;2=-2.45\times 10^{14}$; no units).^67^ In the longer term, 1 month after five sessions of anodal right PFC tDCS online training in older adults, a decrease in task-evoked ${\mathrm{HbO}}_{2}$ change in the bilateral PFC region was again observed.^59^ Decrease in PFC activation correlated with improved task performance regardless of 1 mA, 2 mA, or sham stimulation.

Conversely, Herrmann et al.^72^ revealed an increase in ${\mathrm{HbO}}_{2}$ and decrease in HHb in the frontotemporal cortex during 1-mA bilateral dlPFC stimulation, regardless of polarity, with a verbal fluency task compared to a control task (VFT mean HHb=−19.7±17.9  mmol×mm versus control task mean HHb=9.9±5.6  mmol×mm; p<0.001). However, this decrease was also observed in the sham group with no between-group differences during the verbal fluency task (active mean HHb=−19.7±17.9  mmol×mm versus sham mean HHb=−11.9±14.5  mmol×mm; p=0.14). An additional study demonstrated a 10% increase in ${\mathrm{HbO}}_{2}$ (0.5  μM) in the ipsilateral frontal cortex compared to baseline during 1-mA anodal left frontal stimulation and an 11% increase with cathodal stimulation.^54^

Choe et al.^52^ carried out 2-mA anodal HD stimulation to the right dlPFC and left M1 with flight simulator and WM tasks and observed similar reductions in ${\mathrm{HbO}}_{2}$ in the corresponding locations. With M1 stimulation, a reduction in ${\mathrm{HbO}}_{2}$ (day 1=0.00024  mM versus day 4=−0.000084  mM) and HHb (day 1=−0.00019  mM versus day 4=−0.00049  mM) was observed in the M1 region during an easy-landing task over a 4-day period (compared to an increase in both with sham M1 stimulation). During the N-back task, M1 stimulation elicited a reduction in ${\mathrm{HbO}}_{2}$ (day 1=0.00015  mM versus day 4=−0.00031  mM) in the dlPFC region, a finding not observed in the M1 region or in any sham stimulation. PFC stimulation reduced ${\mathrm{HbO}}_{2}$ in both regions during the easy-landing task, a finding also observed in sham stimulation but to a smaller magnitude.

##### Offline stimulation

Conversely, following offline stimulation (tDCS prior to task) in two studies, an increase in ${\mathrm{HbO}}_{2}$ was observed.^58^^,^^60^ Comparing 1.5-mA left anodal PFC stimulation to baseline revealed an increase in ${\mathrm{HbO}}_{2}$ in the left PFC (pre=1.206 versus Post=1.307, unknown units).^58^ Compared to sham, 1-mA anodal tDCS to left Broca’s area led to an increase in activation of the left frontal cortex while cathodal stimulation led to a trend toward a decrease in activation.^60^

It should be noted that these studies suffer considerable methodological heterogeneity making it difficult to draw definitive conclusions. For example, despite all including a sham group, exposure to sham could be either prior to active stimulation,^48^ or always poststimulation,^54^ or sometimes without a washout period between the two modes.^67^ One study was performed in the elderly population and utilized repeated sessions.^59^ Furthermore, there was variation in the tasks implemented between and within studies along with a noticeable difference in the time lag for poststimulation fNIRS measurement periods and a lack of reporting for all cortical areas measured.

### 3.6 Use of Combined tDCS and fNIRS in Clinical Disease

A total of six articles (Table 4) combined tDCS/fNIRS in potential clinical applications: ischemic stroke survivors^53^^,^^55^ poststroke depression,^62^ schizophrenia,^61^ nicotine dependence,^68^ and tinnitus.^63^ Almost all of the studies applied tDCS to the prefrontal cortex with only one^53^ placing the stimulation electrode at Cz to focus on assessing neurovascular coupling model. Tasks were implemented in four of the studies to assess the clinical impact of tDCS on cravings with cigarette cue-exposure in nicotine dependence,^68^ psychosis scores in schizophrenia,^61^ cognitive task reaction times in poststroke depression,^62^ and auditory function in tinnitus.^63^

**Table 4:** Studies investigating the effects of tDCS on fNIRS-measured response in diseased population. WM, working memory; PFC, prefrontal cortex; dlPFC, dorsolateral prefrontal cortex; M1, primary motor cortex; SOR, supraorbital region; SMC, sensorimotor cortex; L, left; R, right.

Reference	N	Disease	Stimulation protocol	Task	Concurrent tDCS/fNIRS montage	tDCS parameters	Stimulation area	fNIRS parameters	Measurement area	Results
Dutta et al., 2015^53^	4	Ischemic stroke survivors	Active	—	✓	0.5 mA, $0.053\;\mathrm{mA}/{\mathrm{cm}}^{2}$, 15×30 s	Cz + L-SOR	4 channels, 1 emitter, 4 detectors	Cz	Initial ↓ in ${\mathrm{HbO}}_{2}$.
										Figures suggest ↑ ${\mathrm{HbO}}_{2}$ and ↓ HHb thereafter
Jindal et al., 2015^55^	5	Ischemic stroke survivors	Active	—	✓	$0.053\;\mathrm{mA}/{\mathrm{cm}}^{2}$, 15×30 s	Bilateral PFC + Cz	2 emitters, 2 detectors	Bifrontal cortex	↓ ${\mathrm{HbO}}_{2}$ in first 10 s of each tDCS session versus no stimulation breaks
Kroczek et al., 2016^68^	25	Nicotine dependence	Active/sham (parallel)	Cigarette cue exposure	✓	2 mA, $0.057\;\mathrm{mA}/{\mathrm{cm}}^{2}$, 15 min	L-PFC + R-SOR	13 emitters, 12 detectors	Bilateral PFC	↓ HHb (increased activation) in the L dlPFC with sham stimulation
										↑ connectivity of OFC and dlPFC with active tDCS
Narita et al., 2018^61^	28	Schizophrenia	Active (two sessions/day, 5 consecutive days)	Verbal fluency tasks	—	2 mA, $0.057\;\mathrm{mA}/{\mathrm{cm}}^{2}$, 20 min	L-PFC + R-SOR	52 channels	Bilateral PFC	No comments on Hb changes
										Negative correlation between ↑ in ${\mathrm{HbO}}_{2}$ and ↓ in psychosis score after tDCS
Li et al., 2019^62^	22	Poststroke depression	Active/sham (parallel; five sessions/week, 4 weeks)	Emotional, WM tasks	—	2 mA, 20 min	Bilateral PFC	20 channels, 8 emitters, 7 detectors	Bilateral PFC	Emotion: ↑ ${\mathrm{HbO}}_{2}$ in the PFC post tDCS versus baseline when negative faces presented
										WM: ↑ ${\mathrm{HbO}}_{2}$ in L PFC post-tDCS versus baseline
										↑ ${\mathrm{HbO}}_{2}$ in R PFC post-tDCS versus sham
Verma et al., 2019^63^	1	Chronic tinnitus	Active (two sessions/day, total 20 sessions)	Auditory task	—	2 mA, $0.044\;\mathrm{mA}/{\mathrm{cm}}^{2}$, 20 min	R-PFC + R temporal lobe	20 channels, 8 emitters, 8 detectors	Bilateral temporal lobes (auditory cortex)	↑ ${\mathrm{HbO}}_{2}$ bilaterally after tDCS versus baseline
										↓ intensity of the hyperactivated areas

In ischemic stroke survivors,^53^^,^^55^ tDCS was alternated between on and off epochs for 30 –s each and repeated 15 times targeting either Cz^53^ or the left or right PFC.^55^ This stimulation protocol elicited an initial dip in ${\mathrm{HbO}}_{2}$ in the stimulated regions compared to the off periods. Graphical representations^53^ appear to suggest that ${\mathrm{HbO}}_{2}$ subsequently increased with a decrease in HHb, but there is little to no mention of hemodynamic responses following this initial dip in either study.^53^^,^^55^ Kroczek et al.^68^ reported increased functional connectivity between the left dlPFC and the orbitofrontal cortex (OFC) in subjects with nicotine dependence exposed to smoking cue with 2-mA anodal left PFC tDCS compared to sham. However, there was no difference in craving ratings between two groups and sham stimulation actually increased cortical activation through decreased HHb in the left dlPFC (tDCS=0.005975  mm*mmol/L; sham=−0.019425  mm*mmol/L).

The remaining studies examined the impact of multiple sessions of tDCS on patients with poststroke depression,^62^ schizophrenia,^61^ and tinnitus.^63^ After 20 sessions of 2-mA bilateral dlPFC tDCS (anode left and cathode right) spanning 4 weeks, Li et al.^62^ recorded greater ${\mathrm{HbO}}_{2}$ in the bilateral PFC during emotional judgment and WM tasks compared to baseline, a finding not observed in the sham group. In the right PFC, this increase was greater than the sham group. The tDCS group was also observed to have improved reaction time scores in both tasks following treatment, although there was no obvious assessment of depressive symptoms within this study. Narita et al.^61^ performed 10 sessions of 2-mA anodal left dlPFC tDCS in schizophrenia patients and detected a negative correlation between an increase ${\mathrm{HbO}}_{2}$ (e.g., representative channel 10 mean pre=0.0396 versus Mean post=0.0479, unknown units) in left temporoparietal regions and a decrease in positive and negative syndrome scale psychosis score. Verma et al.^63^ applied 20 sessions of 2-mA anodal right tDCS to the dlPFC of one patient with chronic tinnitus and observed an increase in ${\mathrm{HbO}}_{2}$ across bilateral temporal regions ($\mathrm{pre}=-5.98\times 10^{-6}$ versus $\text{Post}=-4.68\times 10^{-7}$, unknown units) alongside an improved tinnitus handicap (THI) score.

### 3.7 Quality Scoring

Table 5 summarizes the results of Jadad quality scoring. Full quality assessment was deemed appropriate for the 16 studies that utilized a sham-control group. Randomization was used in 63% of these studies but only 19% explained suitable methods of random sequence generation. Only 31% utilized a double-blind approach and half of the studies reported withdrawals/dropouts. As described previously, for three studies,^69^^–^^71^ a reduced scoring system was applied, and only one^71^ utilized randomization and reported on dropouts. These results demonstrate that the studies included in this review were not always of optimal quality. With less than two-thirds reporting randomization and less than one-third reporting a double-blind approach, the risk of selection, detection, and performance biases are increased within these experiments. Furthermore, it was noticeable that a number of studies failed to report on raw data, which is a parameter not included within the Jadad score. This makes it difficult to gain an appreciation of the magnitude of fNIRS responses and whether these align between studies. In the future, studies should aim to utilize a randomized, double-blind approach where possible and report on data to aid understanding and interpretation of findings.

**Table 5:** Total Jadad scores for 19 studies deemed suitable for quality scoring. Higher scores represent higher quality with maximum score of 5.

Reference	Jadad score
Merzagora et al., 2010^56^	1
Takai et al., 2016^65^	1
Muthalib et al., 2018^51^	2
aCao et al., 2018^70^	0
aCao and Liu, 2018^69^	0
aKhan, 2013^71^	2
Muthalib et al., 2013^66^	1
Radel et al., 2017^46^	5
Besson et al., 2019^47^	2
Jones et al., 2015^58^	0
McKendrick et al., 2015^48^	1
Stephens and Berryhill, 2016^59^	2
Ehlis et al., 2016^60^	2
Herrmann et al., 2017^72^	3
Borragan et al., 2018^67^	1
Giovannella et al., 2018^54^	1
Choe et al., 2016^52^	3
Kroczek et al., 2016^68^	3
Li et al., 2019^62^	3

a Non sham-controlled study and therefore maximum score of 3.

## 4 Discussion

This review provides a current state-of-art assessment of the impact of tDCS on fNIRS associated hemoglobin changes in healthy adults and patients. At rest, tDCS was observed to increase cortical activation while task-evoked responses tended toward reduced activation during online stimulation and increased activation following stimulation.

At rest, tDCS was observed to be associated with increases in cortical ${\mathrm{HbO}}_{2}$ change particularly when responses were captured in close proximity to the site of anodal stimulation,^49^^–^^51^^,^^56^^,^^57^ which is in keeping with studies using alternative stimulation and imaging modalities. For example, Polania et al.^73^ combined fMRI with tDCS to demonstrate increased functional coupling between neighboring stimulated regions with a decrease in direct functional connections to distant regions. Correspondingly, Zheng et al.^34^ utilized arterial spin labeling with tDCS to demonstrate a 17% increase in cerebral blood flow during anodal stimulation. PET scanning has demonstrated similar findings with widespread increases in regional cerebral activation.^35^ The impact of TMS on fNIRS responses was reviewed by Curtin et al.,^74^ in which a number of studies cited demonstrated increased ${\mathrm{HbO}}_{2}$ with TMS, a finding again confirmed in PET scanning.^75^ The increase in ${\mathrm{HbO}}_{2}$ is generally thought to be due to an indirect “metabolic hypothesis” whereby an increase in neuronal activation results in additional energy and oxygen consumption, which may explain the brief initial drop in ${\mathrm{HbO}}_{2}$ recorded in some studies.^49^^,^^51^ A range of postulated mediators^76^ then send feedback to vasculature to prompt vasodilation and causes the resultant increase in HbO. An alternative direct “neurogenic hypothesis” states that the increase in ${\mathrm{HbO}}_{2}$ is in direct response to neurotransmitters and neuropeptides causing release of vasoactive mediators with subsequent vasodilation.^77^ This redistribution of blood flow could in turn explain why in contralateral or remote brain regions, neural activation is observed to decrease^65^^,^^69^^,^^70^ or be unchanged.^64^ Blood flow directed toward the reinforced stimulated brain regions can alter neuronal transmission and reduce the synchrony of low-frequency fluctuations. These fluctuations are a representation of functionally related brain regions and hence reduce connectivity in these distant brain regions observed in certain studies.^64^^,^^65^^,^^69^^,^^70^

Regarding task-evoked responses, an overall reduced cortical activation was observed during online stimulation.^45^^–^^48^^,^^52^ Although Muthalib et al.^45^ demonstrated an increase in ${\mathrm{HbO}}_{2}$ during task-evoked stimulation, the authors suggest that may be due to increased skin blood flow rather than cortical hemodynamics per se, and that HHb is a better marker for the latter as it is less susceptible to skin blood flow changes. Nevertheless, Khan^71^ observed increased activation under the anodal electrode even after incorporation of short channel separation, although this study only had a sample size of eight, did not utilize a sham comparison group, did not comment specifically on ${\mathrm{HbO}}_{2}$ changes, and failed to include any comment on other Hb differentials. This aligns with a previous study combining tDCS with MRI, which produced a decrease in blood oxygen level dependent imaging activation in the SMA with M1 stimulation during a motor task.^33^ Similarly, tDCS^78^ and TMS^79^ have been observed to reduce motor cortex excitability during a motor task. This is hypothesized to be due to an increase in neural efficiency of synaptic transmission with a reduction in input required for the same level of neural output. This is perhaps reflected in EEG findings, which revealed an increase in synchronization and therefore strengthened functional connections in stimulated cortical regions.^39^ It is conceivable that attenuated PFC cortical hemodynamic responses reflect a certain offload of attention and curtail the burden associated with cognitively demanding tasks. As per evidence that demonstrates that psychological interventions may influence attention via PFC modulation,^80^^,^^81^ tDCS may exert a similar effect, although the precise neuronal mechanisms remain unclear.

In the immediate period following online stimulation, cortical hemodynamics demonstrated increased cortical activation,^47^^,^^67^ suggested to be due to the increase in blood flow required for motor memory consolidation, although this does appear to decline over time.^59^^,^^67^ Offline anodal stimulation demonstrated an increase in cortical activation in three cognitive studies^54^^,^^58^^,^^60^ and two motor studies,^47^^,^^66^ although the latter was not significant compared to sham. Evidence suggests different neurophysiological mechanisms may be responsible for online and offline effects,^27^^–^^29^^,^^31^ which may explain the different activation patterns demonstrated in this study. In addition, as tDCS was effectively being administered at rest (i.e., prior to task), it could be that the increase in cortical activation is in keeping with ongoing and continued effects observed in the studies that measured fNIRS responses at rest. The correlation between these findings and behavioral responses would aid interpretation of the former, but the majority of task-related studies included in this review report either no improvement of performance or the task was used to simply elicit task-evoked responses rather than as a measure of improved performance outcomes with stimulation.

The combination of fNIRS and tDCS in the patient population is limited to six studies across five medical conditions, which makes it difficult to draw conclusions. However, tDCS does appear to prompt increases in ${\mathrm{HbO}}_{2}$ across stimulated brain regions that are of particular significance in stroke survivors. In this cohort of patients, it is well documented that, after initial blunting of fNIRS responses, motor recovery is associated with a return of more typical hemodynamic patterns.^82^ It is possible that this is supported with tDCS, which could then improve motor recovery.^83^ In addition, depression has been theorized as a failure in recruitment of prefrontal cognitive resources,^84^ and the increased activation observed following tDCS could account for the improvement in clinical outcomes. An overall increase in ${\mathrm{HbO}}_{2}$ was also observed in the remaining patient studies, and symptoms of the various conditions improved especially following repeated sessions of tDCS.^61^^–^^63^ While these findings are promising, the small number of studies per medical condition necessitates much more research with greater sample sizes before definitive conclusions are drawn about the effectiveness of tDCS as a treatment modality for these pathological conditions.

### 4.1 Future Considerations

Currently, tDCS and fNIRS are combined in experimental settings at rest to investigate localized and distant hemodynamic correlates of electrical fields generated by various tDCS electrode montages and stimulation protocols. Furthermore, we have discussed the use of combined tDCS and fNIRS in revealing task-evoked activation patterns during a range of online and offline motor and cognitive tasks. For studies related to clinical disease, the technology is being utilized to assess the changes in cortical hemodynamics in ischemic areas; the long-term changes following repetitive tDCS in the case of neuropsychiatric disease. It is envisaged that this combination of technologies will shed further light on the underlying neural mechanisms of tDCS in such disease-related settings. In addition, it may facilitate the precision in the choice of stimulation parameters required to achieve the desired neurophysiological effect. The mobility and relative ease of use of these technologies allow them to be employed in naturalistic environments. For example, tDCS has been used to enhance performance in high cognitive load environments in the military^85^^,^^86^ and surgery.^87^^,^^88^ In these aforementioned applications, if fNIRS is combined with tDCS, a powerful tool could be established to elucidate the physiological impact of tDCS in the real-world settings and would be a step forward to transition the conventional neurophysiological studies from the laboratory to naturalistic environments.

As outlined previously, there is considerable heterogeneity of the setups used to conduct tDCS and fNIRS simultaneously. Currently, a common approach is to utilize commercially available compatible systems for integration, e.g., Starstim (Neuroelectrics, Barcelona, Spain) with Oxymon Mk III (Artinis Medical Systems, Zetten, Netherlands). Several laboratories have also developed combined tDCS and fNIRS systems, which might be cost-effective when compared to commercial ready-integrated systems. Through assessment of integration strategies used by different research groups, the characteristics of an ideal tDCS and fNIRS combination can be postulated. The use of popular high fidelity tDCS stimulation devices and fNIRS optical systems would ensure accurate delivery of stimulation and generate precise electrical fields, followed by acquisition of high quality hemodynamic signals. However, it is crucial to understand hemodynamic changes during the stimulation period itself and therefore we believe that a system that allows concurrent tDCS and fNIRS application would be a richer source of neurophysiological information. In ideal terms, an fNIRS channel should be able to acquire hemodynamic data at the site of stimulation as well as from functionally connected regions. Furthermore, the use of short fNIRS channels is a crucial addition in this setup. Short separation channels (with <10  mm source–detector separation) would allow regress out the increased blood flow changes in the scalp due to warmth and erythema produced underneath the tDCS electrode pads.^89^ Excluding this from cortical fNIRS signals would enable a far more accurate representation of isolated cerebral hemodynamic responses. Comfort is another important aspect to be considered with placing numerous devices on the scalp concurrently. Lightweight, wireless, and ergonomically designed sensor housing for optodes and electrodes would minimize discomfort, e.g., blunt tip or dual-tip optodes (NIRx Medical Technologies, GmbH, Germany; GowerLabs, United Kingdom).

### 4.2 Limitations

One of the major limitations of this review is the lack of objective data reported within the included studies. To overcome this, we included data where reported and additionally contacted all authors for further information. However, the final amount of data we are able to present remains limited, which calls for greater quantitative data reporting in tDCS-fNIRS responses. Furthermore, the high degree of methodological variability makes it challenging to compare and contrast study findings. The works differed in terms of protocol (parallel/crossover), neurostimulation type (conventional tDCS/HD-tDCS, anodal/cathodal tDCS) intensity, duration, number of sessions, and use of sham stimulation as well as neuroimaging parameters, including number of channels, channel locations, and reporting of different hemoglobin subspecies. Moreover, certain investigators developed setups allowing for real-time measurement of cortical activation changes while others could only compare fNIRS results collected pre- and poststimulation. The works selectively presented changes in hemoglobin subspecies concentrations, with most of the studies only depicting ${\mathrm{HbO}}_{2}$ results with few reporting quantitative HHb and HbT data. These methodological and reporting inconsistencies are demonstrated by the generally low-quality scores among studies and limit the scope of comparative analysis of the results. Furthermore, a consistent and major methodological flaw across the majority of studies is the lack of short channel subtraction from hemodynamic changes to account for skin blood flow. Attempts to regress out skin blood flow were made in only four studies,^46^^,^^55^^,^^65^^,^^71^ which suggests that the data presented in many of these investigations could be influenced by skin artifact.

## 5 Conclusion

The combination of tDCS and fNIRS is becoming an increasingly popular and promising technique to investigate neuromodulation and its impact on cortical function. This review highlights several consistent results across the included studies, despite the high degree of methodological heterogeneity and the lack of short channel separation inclusion. Further randomized controlled studies with standardized reporting and higher sample sizes are required to strengthen the evidence of the impact of tDCS on cortical hemodynamics.
